# Giant testicular tumor- a case presentation

**Published:** 2012-09-25

**Authors:** C Grigore, T Poteca, M Forminte, SO Ionescu, S Nedelea

**Affiliations:** * 1st Surgery Department of Colentina Clinical Hospital, Bucharest, Romania; ** 1st Surgery Clinic of “Alexandru Trestioreanu” Institute of Oncology, Bucharest, Romania; *** ”Carol Davila” University of Medicine and Pharmacy, Bucharest, Romania

**Keywords:** seminoma, orchiectomy, acute scrotum

## Abstract

**Background. **Testicular cancer is the most common cancer in men 15 to 35 years old. Histological subtypes are seminoma, non-seminoma and mixed tumours (partly seminoma and partly non-seminoma). Seminomas are more sensitive to radiation therapy and are easier to cure than non-seminomas. The surgical treatment is either orchiectomy, either orchiectomy plus lymph node dissection of the involved ganglia.

**Case presentation. **We present the case of a 42-year-old man with scrotal pain, important swelling and erythema admitted into our surgical unit. Clinical exam and ultrasound revealed a testicular augmentation of 6/15 cm. Radical orchiectomy was performed and the patient was further referred to the oncology department.

**Conclusions.** Even though the common causes of scrotal erythema with local swelling and pain are orchiepididimitis and testicular torsion, a careful examination followed by a precise ultrasound can reveal a developing testicular tumor, which was complicated by inflammation. Moreover, a careful anamnesis hints to the development of a tumor as the patient was operated on for cryptorchidism in childhood. Orchiectomy followed by radiotherapy in seminomas, has a cure rate of 70 to 100%.

## Introduction

Testicular cancer was the first epidemiologically studied disease due to its association with a risk factor. It was noticed that testicular tumors were more frequent in workers involved in chimney sweeping. This was the first occupational hazard described in history. 

Testicular cancer represents the most common malignancy in males aged 15-34 years [**1**]. Histopathologically, testicular germ cell tumors are divided into two major groups: pure seminoma and nonseminoma. The pathogenesis of testicular germ cell tumors remains unknown; however, although recently questioned [**2**], cryptorchidism is the main risk factor, and molecular studies have shown strong evidence of an association between genetic alterations and testicular germ cell tumors [**3**]. 

Nearly 40% of the cases correspond to seminomas and three quarters of them are diagnosed with stage I of the disease [**1**]. Although testicular cancer has excellent cure rates, the choice of treatment centre is of utmost importance. Expert centres achieve better results for both the early stage testicular cancer (lower relapse rates) and overall survival (higher stages within clinical trials) [**4**].

Any testicular or scrotal mass should be considered neoplastic until proven otherwise [**5**]. Seminomas usually preserve testicular shape, while teratomas modify it. A small nodule in the testis represents the initial phase, in which curability rates are the highest. 

Testicular tumor most often presents as a painless enlargement and induration [**5**]. Acute scrotum (tumor, rubor, dolor, calor) represents a rare presentation of testicular cancer and poses differential diagnosis dilemmas, especially regarding orchiepididymitis.

## Case presentation

A 42-year-old patient was admitted for a 1-year history of left scrotal swelling, recently complicated by local hyperemia and pain. The patient had a history of cryptorchidism for which he had surgery in childhood.

Clinical examination was non-specific except for a 6/15 cm, hard, intensely painful on palpation, left testicular tumor with inflamed adjacent tissues.

Basic blood work showed further signs of inflammation with a white cell count of 16500/dl, a high sedimentation rate (74mm/h) and a fibrinogen level of 1084mg/dl. The patient’s alkaline phosphatase had values of 937UI/l. Structural modifications of the left testis revealed by ultrasonography along with clinical and paraclinical data were characteristic for testicular malignancy. There were no regional adenopathies present on ultrasound examination.

We decided to perform a left orchiectomy that had no intra or postoperative complications. Histopathologic examination showed cribriform and pseudoglandular pattern seminoma, with T2N0 stage stadialization.

**Fig. 1:**
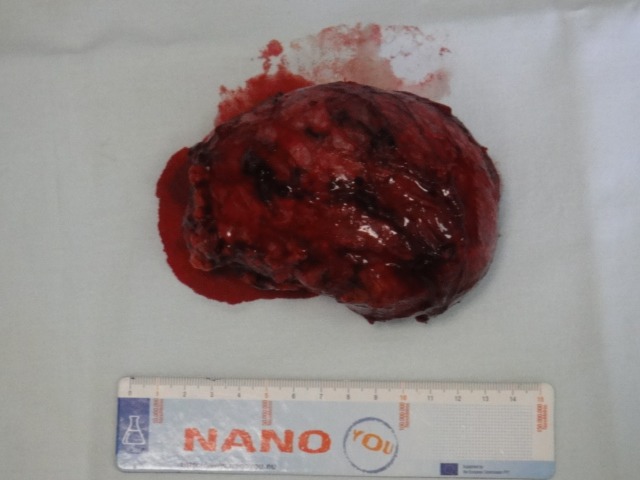
Macroscopic view of resected specimen

**Fig. 2:**
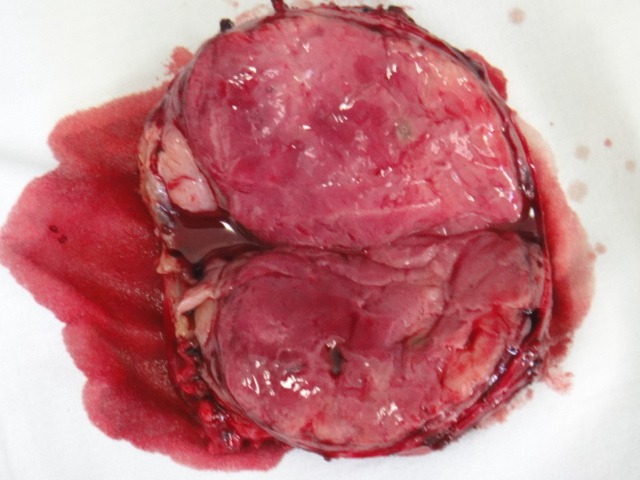
Macroscopic view of resected specimen (section)

**Fig. 3:**
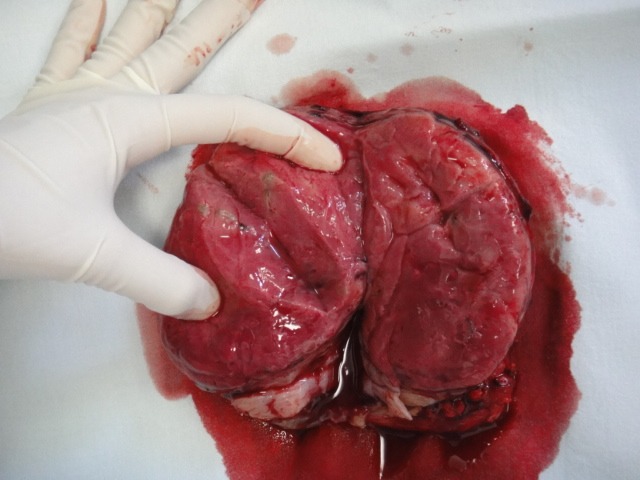
Macroscopic view of resected specimen (section)

Postoperatively, the patient evolved uneventfully both locally and generally and was referred to an oncology department for further treatment. 

## Discussion

Our patient’s history suggests the association between cryptorchidism and testicular malignancy development, although the condition was surgically corrected before the age of one. Delayed presentation was not due to limited access to appropriate medical facilities as cited in other studies [**6**] but to the patient’s ignorance of the massive scrotal swelling that had been persisting for at least 12 months. In this case, the onset of acute scrotum might even be considered providential.

Although the clinical examination did not lead to the final diagnosis, the increased alkaline phosphatase levels and ultrasound examination, that is an excellent, safe, and reliable method for the evaluation of the patients with scrotal diseases, eliminating the need for unnecessary investigations [**7**], suggested a malignant pathology.

A review of the literature showed that this form of presentation is rare [**8-10**]. Although the recent diagnostic and therapeutic developments have altered the prognosis in this disease, the delay in diagnosis and occasional mismanagement of patients continue to inhibit further improvement in survival rate. A high index of suspicion and an aggressive approach to its management are advocated to improve long-term survival [**11**].

The use of cisplatin-based combination chemotherapy has led to a dramatic improvement in the cure rate of patients with metastatic germ cell tumors (GCTs). High complete response (CR) rates were achieved in approximately 80% of the patients with advanced testicular cancer after standard first-line cisplatin-based chemotherapy. Thereafter, the goals of various trials are to reduce the chemotherapy toxicity by limiting the number of chemotherapy cycles, the duration of therapy, and reducing its doses, or even omitting, individual cytotoxic drugs, while maintaining efficacy [**12**].

## Conclusion

Although rare, testicular tumor should be considered in all male patients presenting with acute scrotum. Measures for the increase of patient awareness regarding genital pathology should be taken in order to lower the period between the onset of symptoms and hospital presentation.
